# Effects of graded prevention intervention combined with minimally invasive restoration on the oral health trajectory of caries-susceptible children

**DOI:** 10.3389/fpubh.2026.1862313

**Published:** 2026-05-28

**Authors:** Xue Bai

**Affiliations:** Harbin Institute of Technology Hospital, Harbin, Heilongjiang, China

**Keywords:** caries-susceptible children, graded prevention, minimally invasive restoration, new caries, oral health trajectory

## Abstract

Caries-susceptible children show progressive oral health decline caused by ecological imbalance, poor saliva function and unhealthy habits. Conventional single-mode interventions fail to deliver precise and sustained control. This prospective, single-center, randomized controlled trial investigated graded prevention plus minimally invasive restoration for improving oral health trajectory in these children, to support precise caries control. A total of 186 caries-susceptible children aged 3–6 years were randomly assigned to observation and control groups (*n* = 93 each). The control group received routine oral care, fluoridation and traditional fillings. The observation group received risk-stratified prevention and minimally invasive restoration. All children were followed up for 12 months. Oral reexaminations were performed at 1, 3, 6, and 12 months after intervention, and longitudinal data were analyzed using repeated-measures ANOVA. After follow-up, the observation group had significantly lower dmfs, PLI and SBI (*p* < 0.01), and a much lower new caries rate (8.60% vs. 32.26%, *p* < 0.05). Stratified analysis confirmed better effects in 5–6-year-olds and high-risk children. Multivariate Logistic regression adjusted for socioeconomic status, caregiver education level, daily sugar intake frequency, toothbrushing frequency, and mouth breathing showed that the combined regimen and good oral hygiene were protective factors, while high caries risk, frequent sugar intake, and mouth breathing were risk factors. The 12-month restoration retention rate was also higher (96.77% vs. 82.80%, *p* < 0.05). This combined approach effectively delays caries, improves periodontal health, lowers new caries risk and enhances retention, especially in 5–6-year-old and high-risk children. This combined regimen effectively reshapes the oral health trajectory and may be cautiously applied in clinical practice for precise prevention and control of childhood caries.

## 1 Introduction

Childhood caries, a highly prevalent chronic infectious oral disease worldwide, has been listed by the World Health Organization as one of the major public health problems affecting children’s physical and mental health. Its high incidence, high concealment, and high recurrence rate pose a serious threat to children’s oral health and even systemic growth and development (1). In China, the prevalence of deciduous tooth caries in 5-year-old children has reached 70.9%, and the rate is significantly higher in rural areas than in urban areas, while the caries filling rate is only 4.1%, indicating a severe prevention and control situation (2). Among them, caries-susceptible children, as the core population with high caries incidence, show a continuous deterioration trend in oral health trajectory due to multiple factors such as oral microecological imbalance, abnormal saliva secretion, low mineralization degree of deciduous teeth, and poor diet and oral hygiene habits, as well as mouth breathing, making them the focus and difficulty of childhood caries prevention and control (3).

The deterioration of oral health in caries-susceptible children presents clear multi-factor synergistic pathogenic characteristics, and its core mechanism lies in the interaction between oral microecological homeostasis imbalance and decreased host defense ability (4). Under normal physiological conditions, the microbial community in children’s oral cavity is in a dynamic balance. However, in caries-susceptible children, cariogenic bacteria such as *Streptococcus mutans* and Lactobacillus proliferate in large quantities, breaking the homeostasis of dental plaque biofilm, leading to abnormal carbohydrate metabolism and a continuous decrease in tooth surface pH, and ultimately causing demineralization of dental hard tissues and caries formation (5). Meanwhile, most of these children suffer from problems such as reduced saliva secretion and weakened buffering capacity. In addition, preschool children aged 3–6 years have insufficient self-oral cleaning ability, their parents lack oral health awareness, and they frequently intake high-sugar foods. Mouth breathing further aggravates oral dryness and cariogenic risk. These adverse behaviors further aggravate the occurrence and progression of caries, even leading to pulpitis and premature tooth loss, affecting jaw development and permanent tooth eruption (6).

At present, the prevention and control of childhood caries still adopts the traditional single intervention mode, mainly including routine oral health education, topical fluoridation, and traditional filling restoration. However, such a mode has obvious limitations and fails to achieve precise management and long-term maintenance of oral health in caries-susceptible children (7). Traditional preventive measures lack pertinence and do not implement personalized intervention according to children’s caries risk level, resulting in limited prevention and control effects on children at high caries risk. Traditional restoration techniques mostly use amalgam and conventional resin composite filling, which have problems such as large trauma in caries removal, insufficient retention of dental tissue, and low long-term retention rate of restorations. These techniques cannot effectively block the progression of caries, and may even aggravate children’s fear of oral treatment due to traumatic stimulation, affecting intervention compliance (8).

With the continuous development of precision and minimally invasive concepts in stomatology, graded prevention intervention and minimally invasive restoration techniques have been gradually applied in the field of childhood caries prevention and control (9). Graded prevention intervention is based on caries risk assessment and implements stratified management according to children’s caries susceptibility, realizing personalized and precise preventive measures. Minimally invasive restoration techniques emphasize maximizing the retention of healthy dental tissue, and can effectively block the progression of caries and improve restoration effects by means of minimally invasive caries removal, resin infiltration sealing, silver diamine fluoride minimally invasive restoration, etc., while reducing tissue trauma (10). However, studies on the long-term impact of the combined application of the two on the oral health trajectory of caries-susceptible children are still scarce, especially stratified analysis for children of different ages and caries risk levels, making it difficult to clarify the applicable population and clinical value of the combined regimen.

Based on the above research background, this study focuses on the precise regulation of the oral health trajectory of caries-susceptible children, explores the application effect of graded prevention intervention combined with minimally invasive restoration techniques, and analyzes the intervention differences in different stratified populations and the influencing factors of new caries. The aim is to provide high-quality evidence for the precise prevention and control of childhood caries, optimize prevention and control strategies, realize the positive reshaping of the oral health trajectory of caries-susceptible children, and support the development of children’s oral health.

## 2 Materials and methods

### 2.1 Participants

This prospective, single-center, randomized controlled trial enrolled a total of 186 caries-susceptible children aged 3–6 years who visited and received physical examination in the Department of Stomatology, School Hospital of Harbin Institute of Technology from January 2021 to January 2024 were enrolled. The study covered age-appropriate children in the urban area where the author’s unit is located in Harbin. This study has been approved by the Ethics Committee of the Second Affiliated Hospital of Harbin Medical University. All guardians of the children have given informed consent and signed written informed consent forms.

Sample size was calculated using new caries incidence as the primary endpoint. A new caries rate of 30% in the control group and 10% in the observation group was assumed, with type I error *α* = 0.05 and test power = 0.90. The minimum required sample size was 87 children per group. A total of 93 children per group were enrolled to account for possible attrition during follow-up.

Inclusion Criteria: (1) Children aged 3–6 years, with no gender restriction. (2) Met the definition of caries-susceptible children: at least 1 new carious tooth in the past 1 year, or obvious plaque accumulation, poor feeding, diet and oral hygiene habits, and confirmed as caries-susceptible by oral caries risk assessment. (3) Able to cooperate with oral examination and follow-up, and guardians could comply with intervention and re-examination as required. (4) No severe systemic diseases, congenital oral malformations or mental and behavioral abnormalities.

Exclusion Criteria: (1) Severe systemic diseases such as liver and kidney dysfunction, immunodeficiency diseases or hematological diseases. (2) Allergy to fluoride preparations, resin materials, silver diamine fluoride or other research-related drugs and restorative materials. (3) Received other professional oral preventive interventions or caries restorative treatments in the past 3 months. (4) Severe dental developmental abnormalities, maxillofacial malformations or acute oral infectious diseases. (5) Unable to complete the whole follow-up or with incomplete data.

A total of 186 caries-susceptible children were randomly divided into observation group and control group using a random number table generated by a third-party statistician, with allocation concealment using sealed opaque envelopes. Outcome assessors were blinded to group allocation, while operators and examiners were not blinded due to the nature of interventions. There were no post-randomization exclusions. All 186 children completed the 12-month follow-up (100% retention rate), achieved through dedicated oral health files, telephone/WeChat reminders, and caregiver collaborative management. There were no statistically significant differences in general data such as age, gender distribution, body mass index, socioeconomic status, caregiver education level, daily sugar intake frequency, toothbrushing frequency, mouth breathing, oral caries risk level, baseline decayed-missing-filled surfaces (dmfs), plaque index (PLI), and gingival bleeding index (SBI) between the two groups (*p* > 0.05), indicating comparability.

### 2.2 Intervention

#### 2.2.1 Intervention protocol for control group

The control group adopted routine oral prevention + traditional filling restoration protocol.

Routine oral health education: Guide guardians and children on correct brushing methods, reasonable diet control, and reduction of intake of sweets and carbonated drinks through oral explanation combined with picture education.

Topical fluoridation intervention: Professional full-mouth tooth surface fluoridation was performed once every 6 months using 2% sodium fluoride paint, evenly coated on all tooth surfaces, kept dry for 3–5 min, and fasting and water deprivation were required within 30 min after intervention.

Traditional caries restoration: For affected teeth with caries cavities, conventional caries removal was performed using a high-speed turbine, standard cavity preparation was made, and conventional filling restoration was performed with amalgam or resin composite according to the location and depth of caries.

#### 2.2.2 Intervention protocol for observation group

The observation group adopted caries risk graded prevention intervention + minimally invasive restoration technique combined protocol, with specific implementation as follows.

Children were stratified into high, moderate, and low caries risk groups according to the 2019 American Academy of Pediatric Dentistry (AAPD) caries risk assessment system, combined with baseline oral examination, plaque index, saliva flow rate, diet, oral hygiene habits, and mouth breathing. This tool showed good consistency with a Kappa value of 0.82 in a preliminary experiment.

Low caries risk children: Oral examination once every 6 months, enhanced oral hygiene education, topical fluoridation once every 6 months, guidance to establish regular brushing and diet control habits.

Moderate caries risk children: Reexamination once every 3 months, topical fluoridation once every 3 months, increased plaque cleaning guidance, restriction of sugar-containing food frequency, establishment of diet logs.

High caries risk children: Follow-up intervention once a month, standardized fluoridation every 3 months, combined with dental floss cleaning, pit and fissure sealing and other preventive measures, strengthened parental supervision and management, strict control of sweets and night feeding habits.

Minimally invasive caries removal was used for carious teeth to retain healthy dental tissue and avoid extensive caries removal by conventional turbine. Resin infiltration sealing was used for early enamel caries and proximal shallow caries to block caries progression. Silver diamine fluoride was used for minimally invasive antibacterial restoration of teeth near the pulp or unable to completely remove caries, to inhibit cariogenic activity and promote remineralization of demineralized dental tissue, completing minimally invasive dental restoration.

### 2.3 Observation indicators and detection methods

All indicators were detected by 2 professionally trained stomatologists who completed calibration before examination. The Kappa value for caries diagnosis was 0.86, and the ICC value for restoration assessment was 0.91. Inconsistent results were reviewed and confirmed by a third stomatologist.

#### 2.3.1 Caries-related indicators

Decayed-missing-filled surfaces (dmfs): Record the total number of carious, missing, and filled surfaces of deciduous teeth in children to evaluate the severity of caries.

Incidence of new caries: Defined according to ICDASII criteria; the proportion of newly developed enamel or dentin caries during 12 months of follow-up.

Retention rate of restorations: Defined as restorations with complete retention, no fracture, no secondary caries, no marginal leakage, and normal function at 12 months; the proportion of such restorations in the total number of restorations.

#### 2.3.2 Oral and periodontal health indicators

Plaque index (PLI): Scored 0–3 according to Silness-Löwe plaque index standard; higher score indicates more severe plaque accumulation.

Gingival bleeding index (SBI): Scored 0–5 according to Mühleman-Son gingival crevicular bleeding index; higher score indicates more obvious gingival inflammation and bleeding.

### 2.4 Stratification and statistical methods

Stratified by age: 3–4 years group, 5–6 years group.

Stratified by caries risk: high caries risk, moderate caries risk, low caries risk.

The changes of oral health indicators were compared between the two groups under different stratifications.

Exclusive oral health files were established for all children in both groups, with continuous follow-up for 12 months. Oral reexamination was performed at 1, 3, 6, and 12 months after intervention to record caries progression, periodontal health status, restoration status, and new caries occurrence. The observation group completed staged enhanced intervention synchronously according to the grading protocol, and the control group completed regular reexamination according to the routine protocol to ensure complete and valid follow-up data.

SPSS 26.0 statistical software was used for data analysis. Measurement data were expressed as (*x* ± *s*), and longitudinal repeated measurement data were analyzed using repeated-measures ANOVA. Enumeration data were expressed as [*n*(%)], and inter-group comparison was performed using *χ*^2^ test. Multivariate Logistic regression was used to analyze the influencing factors of new caries in caries-susceptible children, adjusted for socioeconomic status, caregiver education level, daily sugar intake frequency, toothbrushing frequency, and mouth breathing. *p* < 0.05 was considered statistically significant.

## 3 Results

### 3.1 Baseline data analysis

A total of 186 caries-susceptible children aged 3–6 years were enrolled and randomly divided into the observation group and the control group, with 93 cases in each group. All subjects completed the 12-month follow-up, with a 100% follow-up rate. There were no significant differences in gender composition, age stratification (3–4 years, 5–6 years), caries risk stratification (high, moderate, low risk), socioeconomic status, caregiver education level, daily sugar intake frequency, toothbrushing frequency, mouth breathing, baseline decayed-missing-filled surfaces (dmfs), plaque index (PLI), or gingival bleeding index (SBI) between the two groups (all *p* > 0.05). The baseline data were well balanced and comparable, as shown in Table 1.

**Table 1:** Baseline characteristics of caries-susceptible children in the two groups.

Characteristic	Observation group (*n* = 93)	Control group (*n* = 93)	*χ*^2^/*t*-value	*p*-value
Male/Female, *n*	50/43	47/46	0.195	0.659
Age stratum, *n*		0.065	0.799	
3–4 years	41	40	–	–
5–6 years	52	53	–	–
Caries risk stratum, *n*		0.086	0.958	
High risk	24	24	–	–
Moderate risk	45	44	–	–
Low risk	24	25	–	–
Socioeconomic status (middle-high /low), *n*	68/25	65/28	0.326	0.568
Caregiver education level (college or above/below college), *n*	70/23	67/26	0.395	0.529
Daily sugar intake frequency (≥3 times / <3 times), *n*	32/61	35/58	0.371	0.542
Daily toothbrushing frequency (≥2 times/<2 times), *n*	69/24	66/27	0.412	0.521
Mouth breathing (yes/no), *n*	11/82	13/80	0.458	0.498
Baseline dmfs, mean ± SD	6.14 ± 1.32	6.09 ± 1.29	0.247	0.805
Baseline PLI, mean ± SD	1.58 ± 0.37	1.56 ± 0.36	0.352	0.725
Baseline SBI, mean ± SD	1.41 ± 0.29	1.39 ± 0.27	0.471	0.638

### 3.2 Comparison of caries and periodontal health indicators between the two groups after intervention

**Figure 1:**
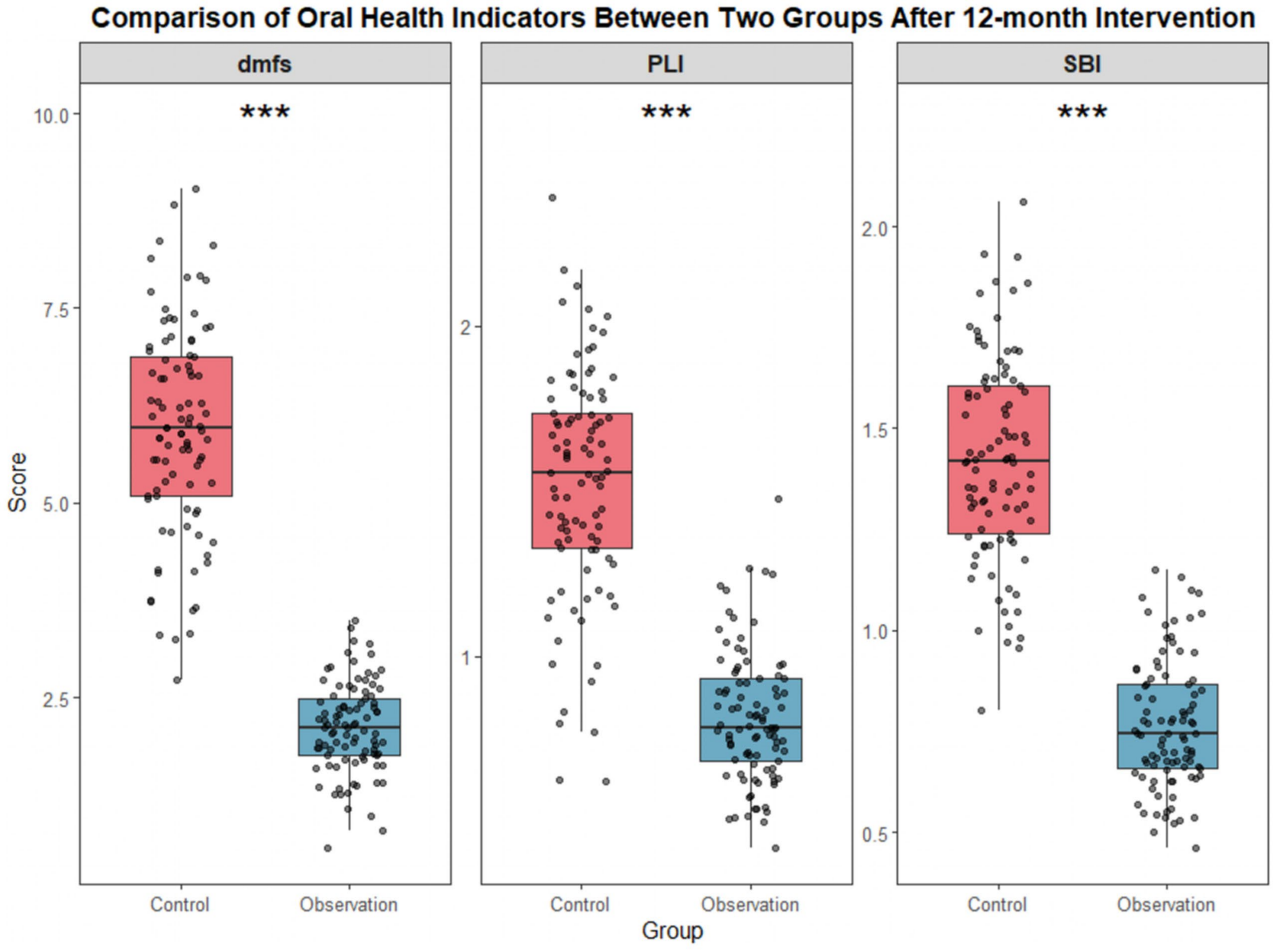
Comparison of dmfs, PLI, and SBI between the two groups after 12-month intervention. Compared with the control group, *p* < 0.01. The abscissa represents groups, and the ordinate represents the score of each indicator. All three indicators in the observation group were significantly lower than those in the control group. ***P < 0.001.

After 12 months of follow-up, dmfs, PLI, and SBI in the observation group were significantly lower than those in the control group, with highly statistically significant differences (all *p* < 0.01), indicating that graded prevention combined with minimally invasive restoration could more effectively control caries progression, improve oral hygiene, and alleviate gingival inflammation, as detailed in Table 2 and Figure 1.

**Table 2:** Comparison of oral health indicators between the two groups after 12-month intervention (*x̄* ± *s*).

Indicator	Observation group (*n* = 93)	Control group (*n* = 93)	*t* value	*p*-value
dmfs	2.07 ± 0.65	5.81 ± 1.25	25.146	<0.001
PLI	0.83 ± 0.20	1.55 ± 0.35	17.923	<0.001
SBI	0.76 ± 0.17	1.38 ± 0.28	18.057	<0.001

### 3.3 Subgroup analysis of age stratification and caries risk stratification

#### 3.3.1 Comparison of oral health indicators in children of different age strata

In the 3–4-year-old subgroup, dmfs, PLI, SBI, and new caries incidence in the observation group were lower than those in the control group, and the restoration retention rate was higher. In the 5–6-year-old subgroup, dmfs, PLI, SBI, and new caries incidence in the observation group were significantly lower than those in the control group, and the restoration retention rate was significantly higher, with highly statistically significant differences (all *p* < 0.001), as presented in Table 3.

**Table 3:** Comparison of oral health indicators between the two groups by age stratification.

Age stratification	Group	*n*	dmfs (*x̄* ± *s*)	PLI (*x̄* ± *s*)	SBI (*x̄* ± *s*)	New caries incidence (%)	Restoration retention rate (%)
3–4 years	Observation group	41	2.31 ± 0.72	1.12 ± 0.35	0.85 ± 0.28	12.20	95.12
	Control group	40	6.17 ± 1.31	1.89 ± 0.42	1.56 ± 0.37	37.50	82.50
	Test value	–	*t* = 15.782	*t* = 9.034	*t* = 9.715	*χ*^2^ = 6.892	*χ*^2^ = 3.973
	*P*-value	–	<0.001	<0.001	<0.001	0.009	0.046
5–6 years	Observation group	52	1.89 ± 0.58	1.05 ± 0.31	0.78 ± 0.25	9.62	96.15
	Control group	53	5.52 ± 1.18	1.95 ± 0.45	1.62 ± 0.41	35.85	83.02
	Test value	–	*t* = 20.419	*t* = 11.876	*t* = 12.633	*χ*^2^ = 10.274	*χ*^2^ = 4.987
	*P*-value	–	<0.001	<0.001	<0.001	0.001	0.026

dmfs, decayed-missing-filled surfaces; PLI, plaque index; SBI, gingival bleeding index. Compared with the control group in the same age stratum, *P* < 0.05 indicates a statistically significant difference, and *P* < 0.001 indicates a highly statistically significant difference.

#### 3.3.2 Comparison of oral health indicators and new caries incidence in children of different caries risk strata

In the high caries risk group, baseline dmfs, PLI, and SBI were similar between the two groups. At 6 and 12 months of intervention, the three indicators in the observation group were significantly lower than those in the control group, and the reduction increased with prolonged intervention, suggesting that combined intervention could effectively control caries progression and improve oral hygiene and gingival inflammation in high-risk children.

In the moderate caries risk group, baseline indicators were comparable between the two groups. Dmfs, PLI, and SBI in the observation group became significantly lower than those in the control group from 6 months, and the difference further widened at 12 months, showing stable intervention efficacy.

In the low caries risk group, no significant differences were observed in all indicators at baseline, 6 months, or 12 months between the two groups, suggesting that the protocol had no additional adverse effects on oral health in low-risk children and favorable safety, as shown in Table 4. The pairwise t‑tests presented in Table 4 were conducted as post‑hoc analyses following repeated‑measures ANOVA, and the overall repeated‑measures ANOVA results were statistically significant.

**Table 4:** Comparison of oral health indicators at each time point between the two groups by caries risk stratification (*x̄* ± s).

Caries risk stratification	Time point	Group	*n*	dmfs	PLI	SBI
High risk	Baseline (0 months)	Observation Group	24	7.25 ± 1.56	2.13 ± 0.42	1.89 ± 0.38
		Control Group	24	7.17 ± 1.49	2.09 ± 0.39	1.85 ± 0.36
		*t* value	–	0.182	0.347	0.375
		*P*-value	–	0.856	0.730	0.709
	6 months	Observation group	24	3.82 ± 0.95	1.25 ± 0.31	0.92 ± 0.27
		Control group	24	6.54 ± 1.32	1.98 ± 0.40	1.76 ± 0.35
		*t* value	–	8.215	7.123	9.468
		*P*-value	–	<0.001	<0.001	<0.001
	12 months	Observation group	24	2.91 ± 0.78	1.08 ± 0.28	0.79 ± 0.24
		Control group	24	6.03 ± 1.27	1.92 ± 0.38	1.68 ± 0.33
		*t* value	–	10.367	8.792	10.854
		*P*-value	–	<0.001	<0.001	<0.001
Moderate risk	Baseline (0 months)	Observation group	45	4.38 ± 1.12	1.85 ± 0.37	1.52 ± 0.32
		Control group	44	4.42 ± 1.09	1.82 ± 0.35	1.49 ± 0.30
		*t* value	–	0.170	0.393	0.458
		*P*-value	–	0.865	0.695	0.648
	6 months	Observation group	45	2.51 ± 0.68	1.19 ± 0.29	0.88 ± 0.25
		Control group	44	4.07 ± 0.98	1.76 ± 0.36	1.43 ± 0.31
		*t* value	–	8.764	8.207	9.215
		*P*-value	–	<0.001	<0.001	<0.001
	12 months	Observation group	45	2.03 ± 0.56	1.05 ± 0.26	0.76 ± 0.22
		Control group	44	3.89 ± 0.92	1.71 ± 0.34	1.38 ± 0.29
		*t* value	–	11.429	10.318	11.376
		*P*-value	–	<0.001	<0.001	<0.001
Low risk	Baseline (0 months)	Observation group	24	1.92 ± 0.65	1.51 ± 0.33	1.18 ± 0.28
		Control group	25	1.88 ± 0.62	1.48 ± 0.31	1.15 ± 0.26
		*t* value	–	0.221	0.332	0.394
		*P*-value	–	0.826	0.741	0.695
	6 months	Observation group	24	1.75 ± 0.58	1.12 ± 0.27	0.85 ± 0.23
		Control group	25	1.81 ± 0.61	1.15 ± 0.29	0.88 ± 0.25
		*t* value	–	0.356	0.378	0.437
		*P*-value	–	0.723	0.707	0.664
	12 months	Observation group	24	1.68 ± 0.53	1.03 ± 0.25	0.77 ± 0.21
		Control group	25	1.74 ± 0.57	1.06 ± 0.27	0.80 ± 0.23
		*t* value	–	0.382	0.405	0.479
		*P*-value	–	0.704	0.687	0.634

dmfs, decayed-missing-filled surfaces; PLI, plaque index; SBI, gingival bleeding index. *P* < 0.05 indicates a statistically significant difference, and *P* < 0.001 indicates a highly statistically significant difference.

**Figure 2:**
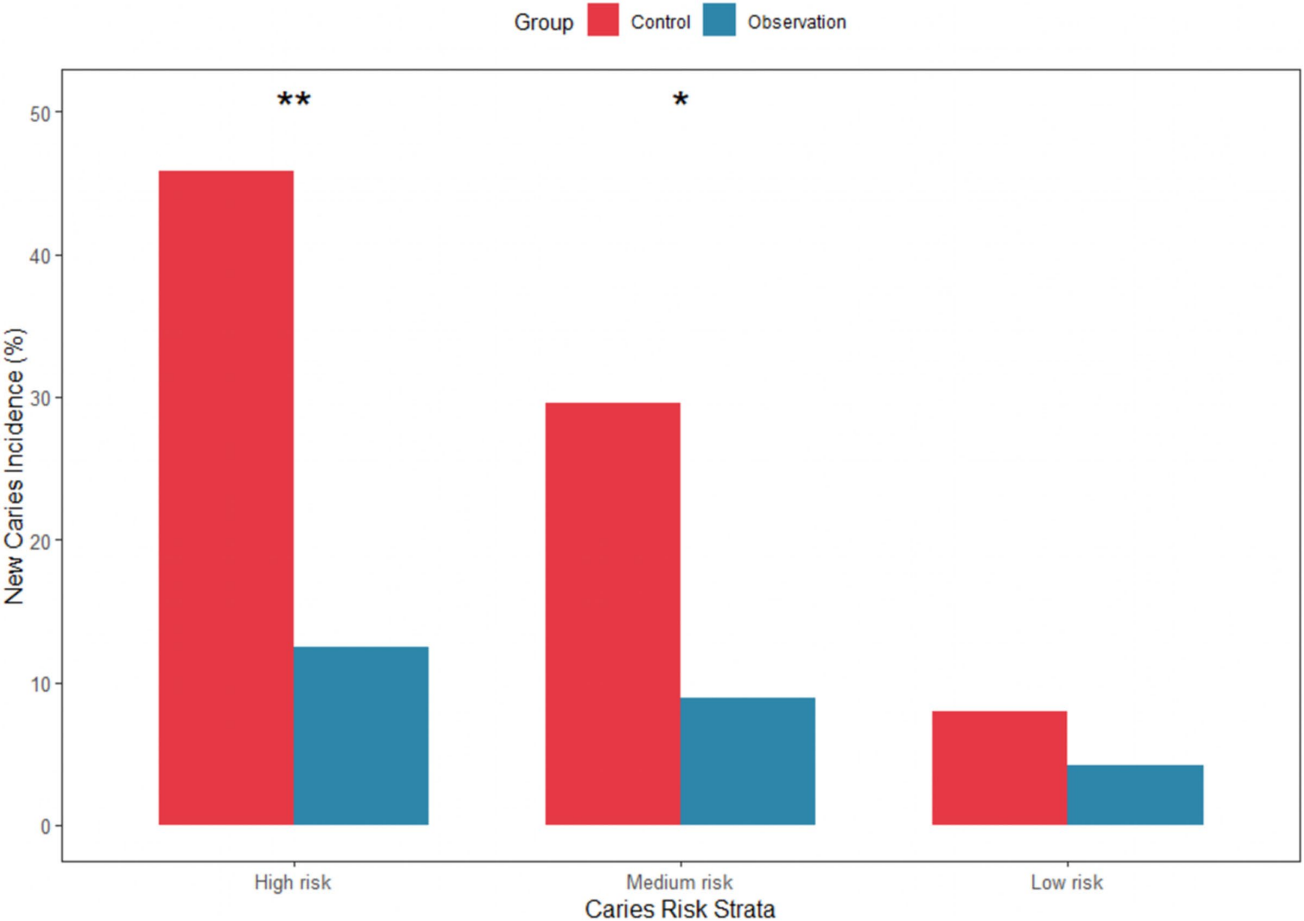
Comparison of new caries incidence between the two groups by caries risk stratification. Compared with the control group in the same stratum, *p < 0.05, **p < 0.01. There was no significant difference between the two groups in the low-risk group.

The new caries incidence in the observation group was significantly lower than that in the control group among children at high caries risk (12.50% vs. 45.83%, *p* < 0.01) and moderate caries risk (*p* < 0.05), whereas no significant difference was found between the two groups in low-risk children (*p* > 0.05), as demonstrated in Table 5 and Figure 2.

**Table 5:** Comparison of new caries incidence between the two groups by caries risk stratification [*n* (%)].

Caries risk stratification	Observation group	Control group	*χ*^2^ value	*P*-value
High risk (*n* = 24/24)	3 (12.50)	11 (45.83)	6.381	0.011
Moderate risk (*n* = 45/44)	4 (8.89)	13 (29.55)	6.514	0.011
Low risk (*n* = 24/25)	1 (4.17)	2 (8.00)	0.000	1.000
Total	8 (8.60)	26 (27.96)	16.329	<0.001

### 3.4 Multivariate logistic regression analysis of factors associated with new caries

Multivariate Logistic regression analysis was performed with new caries as the dependent variable, and intervention mode, age stratification, caries risk level, oral hygiene habits, and fluoridation frequency, socioeconomic status, caregiver education level, daily sugar intake frequency, toothbrushing frequency, mouth breathing as independent variables. The results showed that graded prevention combined with minimally invasive restoration (OR = 0.213, 95%CI: 0.098–0.462, *p* < 0.01), good oral hygiene habits (OR = 0.307, 95%CI: 0.145–0.649, *p* = 0.002), higher socioeconomic status (OR = 0.485, 95%CI: 0.226–0.978, *p* = 0.045), higher caregiver education level (OR = 0.505, 95%CI: 0.231–0.998, *p* = 0.049), and daily toothbrushing ≥2 times (OR = 0.399, 95%CI: 0.179–0.886, *p* = 0.025) served as independent protective factors for new caries. In contrast, high caries risk level (OR = 2.895, 95%CI: 1.368–6.127, *p* = 0.006), daily sugar intake ≥3 times (OR = 3.482, 95%CI: 1.485–8.091, *p* = 0.005), and mouth breathing (OR = 3.018, 95%CI: 1.265–7.214, *p* = 0.013) were identified as independent risk factors. Age stratification was not significantly associated with new caries risk (*p* > 0.05). These findings are summarized in Table 6.

**Table 6:** Multivariate logistic regression analysis of factors associated with new caries in caries-susceptible children.

Factor	*β*	SE	Wald *χ*^2^	OR	95%CI	*P*-value
Graded prevention combined with minimally invasive restoration	−1.547	0.391	15.612	0.213	0.098–0.462	<0.001
Good oral hygiene habits	−1.182	0.382	9.596	0.307	0.145–0.649	0.002
High caries risk level	1.063	0.385	7.623	2.895	1.368–6.127	0.006
Age stratification	−0.215	0.346	0.386	0.807	0.408–1.594	0.534
Socioeconomic status (low as reference)	−0.724	0.412	4.015	0.485	0.226–0.978	0.045
Caregiver education level (low as reference)	−0.683	0.398	3.724	0.505	0.231–0.998	0.049
Daily sugar intake frequency (≥3 times as reference)	1.247	0.436	8.024	3.482	1.485–8.091	0.005
Daily toothbrushing frequency (<2 times as reference)	−0.916	0.407	5.027	0.399	0.179–0.886	0.025
Mouth breathing (yes as reference)	1.105	0.442	6.213	3.018	1.265–7.214	0.013

### 3.5 Comparison of long-term retention of restorations between the two groups

After 12 months of follow-up, the restoration retention rate in the observation group was 96.77%, significantly higher than 82.80% in the control group (*p* < 0.001). This indicates that minimally invasive restoration combined with graded prevention can reduce adverse events such as restoration loss and secondary caries, and improve long-term restoration efficacy, as shown in Table 7.

**Table 7:** Comparison of 12-month restoration retention rate between the two groups.

Group	Total restorations (n)	Retained restorations (n)	Retention rate (%)	*χ*^2^ value	*P*-value
Observation group	93	90	96.77	12.047	<0.001
Control group	93	77	82.80	–	–

## 4 Discussion

The continuous deterioration of the oral health trajectory in caries-susceptible children is mainly attributed to the synergistic effects of oral microecological imbalance, abnormal saliva secretion, mouth breathing, and unhealthy behavioral habits. Traditional interventions mostly adopt routine prevention or restoration strategies, which lack pertinence and fail to achieve long-term management of caries (11). In this study, the observation group received personalized prevention intervention based on caries risk grading combined with minimally invasive restoration techniques, constructing a full-process prevention and control system of “prevention-restoration-maintenance.” As a result, the decayed-missing-filled surfaces (dmfs), plaque index (PLI), and gingival bleeding index (SBI) in the observation group were significantly lower than those in the control group (*p* < 0.01), which confirms the scientificity and effectiveness of the combined regimen (12). Graded prevention intervention implements differentiated prevention and control measures for children at high, moderate, and low caries risk through accurate assessment of caries risk level. High-risk children receive enhanced fluoride application and oral hygiene supervision, while low-to-moderate risk children focus on health education and regular monitoring. This stratified prevention and control model conforms to the core concept of “full-cycle health management” and realizes a paradigm shift from passive response to active intervention (12).

The rational application of minimally invasive restoration techniques is the key support for the efficacy of the combined regimen. Compared with traditional amalgam/resin composite filling in the control group, minimally invasive caries removal, resin infiltration sealing, and silver diamine fluoride minimally invasive restoration used in the observation group have significant advantages in preserving healthy dental tissues, reducing trauma, and blocking caries progression (13). With high fluidity and permeability, resin infiltration can penetrate into the enamel demineralization area, seal micropores, and block acid invasion, effectively inhibiting the progression of early caries, with better efficacy than simple fluoride therapy (14). Silver diamine fluoride can kill cariogenic bacteria, promote dentin remineralization, and reduce the risk of secondary caries; its combined use with glass ionomer can further improve the restoration effect (15). Meanwhile, minimally invasive techniques cause less trauma and discomfort, which can improve children’s treatment compliance and reduce intervention interruption due to fear of treatment. This is also an important reason why the 12-month restoration retention rate in the observation group (96.77%) is significantly higher than that in the control group (82.80%) (16), which is consistent with a network meta-analysis showing that the long-term success rate of minimally invasive restoration techniques is significantly better than that of traditional restoration methods (17).

Stratified analysis shows that the combined regimen has better intervention effect in caries-susceptible children aged 5–6 years than in those aged 3–4 years, and the benefit is more significant in children at high caries risk, which has important clinical guiding significance. Children aged 5–6 years are in the late stage of primary dentition and the early stage of permanent tooth eruption, with stronger oral cleaning ability and better cooperation with intervention implementation than those aged 3–4 years. At the same time, primary caries progresses rapidly at this stage, so the advantage of precise prevention and control of the combined regimen is more prominent (18). However, children aged 3–4 years have low cognitive level, poor oral hygiene compliance, low saliva secretion, and weak buffering capacity. Those with mouth breathing face greater challenges in caries control. Even with intervention, it is difficult to quickly reverse the deteriorating oral health trajectory, suggesting that intervention frequency should be increased and parental collaborative supervision should be strengthened for this age group (19). Children at high caries risk have more severe oral microecological imbalance and high cariogenic bacterial load, and traditional intervention modes are difficult to effectively control caries progression. Graded prevention combined with minimally invasive restoration can control cariogenic factors from the source and rapidly repair lesions, so the incidence of new caries (12.50%) is significantly lower than that in the control group (45.83%) (20). Low-risk children have few cariogenic factors, and routine prevention can meet the prevention and control needs, so there is no significant difference in the incidence of new caries between the two groups (*p* > 0.05). This is consistent with an early systematic review concluding that precise stratified intervention can improve the efficiency of prevention and control resource utilization (21).

Multivariate Logistic regression analysis further confirms that graded prevention combined with minimally invasive restoration, good oral hygiene habits, higher socioeconomic status, higher caregiver education level, and daily toothbrushing ≥2 times are protective factors for reducing new caries in caries-susceptible children, while high caries risk level, frequent sugar intake, and mouth breathing are risk factors (*p* < 0.01). This result profoundly reveals the mechanism influencing the oral health trajectory of caries-susceptible children. The combined intervention cuts off the cariogenic chain through graded prevention and blocks lesion progression via minimally invasive restoration, forming a closed-loop management of “prevention-restoration-consolidation” to fundamentally reduce the risk of new caries (22). Good oral hygiene habits can reduce plaque accumulation and the probability of cariogenic bacterial reproduction, forming a synergistic effect with intervention measures to jointly maintain oral health (23), while insufficient oral hygiene compliance is an important cause of recurrence of childhood caries (24). Children at high caries risk have a high inherent risk of caries due to congenital factors such as abnormal saliva secretion and strong cariogenic colonization ability. Coupled with mouth breathing and high sugar intake, their caries risk increases synergistically. Without precise intervention, their oral health trajectory tends to deteriorate continuously, suggesting that clinical attention should be focused on this group and a stricter personalized prevention and control plan should be formulated (25).

This study still has certain limitations. First, this is a single-center study with limited sample representativeness; future studies should expand the sample size and include children from different regions and socioeconomic backgrounds to verify the generalizability of the combined regimen. Second, the intervention is a combined package, and the independent effect of graded prevention and minimally invasive restoration cannot be separated. Third, the follow-up period is 12 months, and the long-term intervention effect still needs longer follow-up observation. Fourth, the specific mechanism of the combined regimen on oral microecology has not been deeply discussed; subsequent studies can improve the research on intervention mechanism by detecting indicators such as cariogenic bacterial load and saliva buffering capacity.

## 5 Conclusion

Graded prevention intervention combined with minimally invasive restoration significantly improves the oral health status of caries-susceptible children aged 3–6 years. This combined regimen effectively reduces decayed-missing-filled surfaces, plaque accumulation, and gingival inflammation, lowers the incidence of new caries, and improves the 12-month retention rate of restorations. The beneficial effects are more prominent in children aged 5–6 years and those at high caries risk. The combined intervention, good oral hygiene habits, higher socioeconomic status, higher caregiver education level, and adequate toothbrushing are independent protective factors against new caries, while high caries risk, frequent sugar intake, and mouth breathing are independent risk factors. This strategy can positively reshape the oral health trajectory of caries-susceptible children with reliable safety and stable short-term efficacy. This combined approach is worthy of cautious clinical application in the precise prevention and control of childhood caries, and further validation by multi-center, large-sample, long-term follow-up research is recommended.

## Data Availability

The raw data supporting the conclusions of this article will be made available by the authors, without undue reservation.

## References

[1] World Health Organization (2025) Sugars and dental caries [fact sheet]. Available online at: https://www.who.int/news-room/fact-sheets/detail/sugars-and-dental-caries (Accessed March 15, 2026).

[2] Chen J Chen W Lin L Ma H Huang F. The prevalence of dental caries and its associated factors among preschool children in Huizhou, China: a cross-sectional study. Front. Oral Health. (2024) 5:1461959. doi: 10.3389/froh.2024.1461959, 39280639 PMC11392855

[3] Maklennan A Borg-Bartolo R Wierichs RJ Esteves-Oliveira M Campus G. A systematic review and meta-analysis on early-childhood-caries global data. BMC Oral Health. (2024) 24:835. doi: 10.1186/s12903-024-04605-y, 39049051 PMC11267837

[4] Spatafora G Li Y He X Cowan A Tanner ACR. The evolving microbiome of dental caries. Microorganisms. (2024) 12:121. doi: 10.3390/microorganisms12010121, 38257948 PMC10819217

[5] Weng L Cui Y Jian W Zhang Y Pang L Cao Y . Inter-kingdom interactions and environmental influences on the oral microbiome in severe early childhood caries. Microbiol Spectrum. (2025) 13:e02518–24. doi: 10.1128/spectrum.02518-24, 40243315 PMC12131756

[6] Al-Sharani HM Stormon N Al-Hutbany N Zhang Y Zulfiqar T. Optimal tooth brushing initiation age and frequency for preventing early childhood caries: a systematic review and meta-analysis. BMC Oral Health. (2025) 25:2006. doi: 10.1186/s12903-025-07179-5, 41469640 PMC12754868

[7] Jiang HF Shi AT Li J Zhang YH Yang J. Effectiveness of risk-based caries management among Chinese preschool children: a randomized controlled single-blind trial. BMC Oral Health. (2024) 24:673. doi: 10.1186/s12903-024-04442-z, 38851679 PMC11162041

[8] Sahebalam R Ghorbani M Shirazi AS Khosrojerdi M Mowji M. Comparative success of minimally invasive treatments for cavitated caries in primary teeth: a network meta-analysis. BMC Oral Health. (2025) 25:1469. doi: 10.1186/s12903-025-06832-3, 41013342 PMC12465559

[9] BaniHani A Santamaría RM Hu S Maden M Albadri S. Minimal intervention dentistry for managing carious lesions into dentine in primary teeth: an umbrella review. Eur Arch Paediatr Dent. (2022) 23:667–93. doi: 10.1007/s40368-021-00675-6, 34784027 PMC9637620

[10] Tasleem R Alqahtani SA Abogazalah N Almubarak H Riaz A Ali SS . Microinvasive interventions in the management of proximal caries lesions in primary and permanent teeth—systematic review and meta-analysis. BMC Oral Health. (2025) 25:48. doi: 10.1186/s12903-024-05400-5, 39780151 PMC11716243

[11] Yashab J Ayesha N Carpenter F. Role of the early detection and prevention of dental caries in children: a systematic review of clinical outcomes. Cureus. (2025) 17:e85185. doi: 10.7759/cureus.85185, 40600078 PMC12212394

[12] Dong H Zhang Z. Effects of graded preventive measures on dental caries prevention in young children susceptible to caries. Am J Transl Res. (2025) 17:5493–501. doi: 10.62347/UHZX2726, 40821090 PMC12351574

[13] Muntean A Mzoughi SM Pacurar M Candrea S Inchingolo AD Inchingolo AM . Silver diamine fluoride in pediatric dentistry: effectiveness in preventing and arresting dental caries—a systematic review. Children. (2024) 11:499. doi: 10.3390/children11040499, 38671716 PMC11049537

[14] Kelmendi M Robo I Petro E Kelmendi S. Silver diamine fluoride in arresting dental caries among young children: a randomized clinical trial. Med Arch. (2025) 79:399–405. doi: 10.5455/medarh.2025.79.399-405, 41282046 PMC12634075

[15] Devan I Janakiram C Ramanarayanan V Varma BR Vasudevan S. Effectiveness of silver diamine fluoride application at various frequencies for dental caries cessation among preschool children in India: randomized controlled trial. Contemp Clin Dentistry. (2025) 16:28–35. doi: 10.4103/ccd.ccd_535_24, 40270866 PMC12013995

[16] Desai H Stewart CA Finer Y. Minimally invasive therapies for the management of dental caries—a literature review. Dent J. (2021) 9:147. doi: 10.3390/dj9120147, 34940044 PMC8700643

[17] González-Gil D Flores-Fraile J Vera-Rodríguez V Martín-Vacas A López-Marcos J. Comparative meta-analysis of minimally invasive and conventional approaches for caries removal in permanent dentition. Medicina. (2024) 60:402. doi: 10.3390/medicina60030402, 38541128 PMC10971845

[18] Cao P Zhang Y Houser SH Yang J Tao W Zhang F . Evaluating an early childhood caries prevention strategy: a study protocol for a stepped-wedge cluster randomised controlled trial. BMJ Open. (2025) 15:e102143. doi: 10.1136/bmjopen-2025-102143, 40754328 PMC12320031

[19] Molinari AH Grap ME Pierce SL Sauer AG Belay B Goodman AB . Caregiver-reported sugar-sweetened beverage consumption and cavities in children aged 1 to 5 years, National Survey of children’s health 2021–2022. Prev Chronic Dis. (2025) 22:250183. doi: 10.5888/pcd22.250183, 40934341 PMC12447843

[20] Al-Kaff AA Alshehri AZ Alasmari RA Alsubaie N Aldaws A Althaqeel A . Minimally invasive techniques for managing dental caries in children: efficacy, applications, and future directions. Cureus. (2025) 17:e87450. doi: 10.7759/cureus.87450, 40772222 PMC12327548

[21] AlHarbi SG Almushayt AS Bamashmous S Abujamel TS Bamashmous NO. The oral microbiome of children in health and disease—a literature review. Front Oral Health. (2024) 5:1477004. doi: 10.3389/froh.2024.1477004, 39502321 PMC11534731

[22] Zheng FM Yan IG Duangthip D Lo ECM Gao SS Chu CH. Randomized clinical trial on caries prevention of silver diamine fluoride. J Dent Res. (2025) 105:443–50. doi: 10.1177/00220345251363837, 40926346 PMC12957404

[23] Liu Y Zhu J Zhang H Jiang Y Wang H Yu J . Dental caries status and related factors among 5-year-old children in Shanghai. BMC Oral Health. (2024) 24:459. doi: 10.1186/s12903-024-04185-x, 38627729 PMC11020175

[24] Liu S Thearmontree A Chongsuvivatwong V Zhang S Zhang L. Association between parental migration and dental caries of 3–12-year-old children in China: a systematic review and meta-analysis. J Int Oral Health. (2023) 15:409–17. doi: 10.4103/jioh.jioh_89_23

[25] Zou J Du Q Ge L Wang J Wang X Li Y . Expert consensus on early childhood caries management. Int J Oral Sci. (2022) 14:35. doi: 10.1038/s41368-022-00186-0, 35835750 PMC9283525

